# The Influence of Bee Venom Melittin on the Functioning of the Immune System and the Contractile Activity of the Insect Heart—A Preliminary Study

**DOI:** 10.3390/toxins11090494

**Published:** 2019-08-27

**Authors:** Jan Lubawy, Arkadiusz Urbański, Lucyna Mrówczyńska, Eliza Matuszewska, Agata Światły-Błaszkiewicz, Jan Matysiak, Grzegorz Rosiński

**Affiliations:** 1 Department of Animal Physiology and Development, Faculty of Biology, Adam Mickiewicz University in Poznań, 61-614 Poznań, Poland; 2 Department of Cell Biology, Faculty of Biology, Adam Mickiewicz University in Poznań, 61-614 Poznań, Poland; 3 Department of Inorganic and Analytical Chemistry, Poznan University of Medical Sciences, 60-780 Poznan, Poland

**Keywords:** melittin, insect immune system, apoptosis, heart contractility, *Tenebrio molitor*

## Abstract

Melittin (MEL) is a basic polypeptide originally purified from honeybee venom. MEL exhibits a broad spectrum of biological activity. However, almost all studies on MEL activity have been carried out on vertebrate models or cell lines. Recently, due to cheap breeding and the possibility of extrapolating the results of the research to vertebrates, insects have been used for various bioassays and comparative physiological studies. For these reasons, it is valuable to examine the influence of melittin on insect physiology. Here, for the first time, we report the immunotropic and cardiotropic effects of melittin on the beetle *Tenebrio molitor* as a model insect. After melittin injection at 10^−7^ M and 10^−3^ M, the number of apoptotic cells in the haemolymph increased in a dose-dependent manner. The pro-apoptotic action of MEL was likely compensated by increasing the total number of haemocytes. However, the injection of MEL did not cause any changes in the percent of phagocytic haemocytes or in the phenoloxidase activity. In an in vitro bioassay with a semi-isolated *Tenebrio* heart, MEL induced a slight chronotropic-positive effect only at a higher concentration (10^−4^ M). Preliminary results indicated that melittin exerts pleiotropic effects on the functioning of the immune system and the endogenous contractile activity of the heart. Some of the induced responses in *T. molitor* resemble the reactions observed in vertebrate models. Therefore, the *T. molitor* beetle may be a convenient invertebrate model organism for comparative physiological studies and for the identification of new properties and mechanisms of action of melittin and related compounds.

## 1. Introduction

Venomous animals have been a focus of interest for humans for years due to the danger associated with them and the possibility of taking advantage of venom action and utilizing it in medicine [1,2]. Animal venoms are immensely complex mixtures composed of proteins, peptides, biogenic amines, and other substances of low-molecular weight, which are often enzymatically active [3,4,5]. Especially, the great interest is related to the biological activity of proteins isolated from bee (melittin, apamina and tertiapin) and wasp (mastoparan and bradykinin) venoms [2].

Melittin (MEL), a 26-amino-acid amphiphilic polypeptide with a hydrophobic *N*-terminal and a hydrophilic *C*-terminal end, is one of the most important bee venom compounds. MEL is the major active component of apitoxin (honeybee venom), constituting from 40% to 60% of whole dry venom [6]. Melittin has various biological, pharmacological, and toxicological actions, including strong surface activity on cell lipid membranes, and haemolytic, antibacterial, antifungal, and potential anti-tumour activities [6,7,8,9]. Melittin is also known as a membrane pore-forming agent that in a dose-dependent manner interacts with the phospholipid bilayer, and the molecular mechanism of interactions between biomembranes and peptides and proteins can be studied using MEL-biological activity [10,11,12]. When MEL binds to cell membranes, it forms toroid-shaped pores that enable the leakage of molecules of tens of kDa in size. This leakage results in a dose-dependent increase in membrane permeability and cell lysis. As a peptide that forms pores, melittin has been extensively studied from diverse aspects, including its structure, binding properties and mechanisms, and disruption of phospholipid bilayer structure processes (reviewed by Chen, et al. [13]). Due to its fascinating interaction with the lipids of the plasma membrane and its capability to form pores, melittin has the potential to be used as an antimicrobial agent, for cell-selective attack, and for the translocation of materials by increasing the membrane permeability [14,15,16]. Melittin binds to negatively charged membrane surfaces with ease and then disturbs the integrity of phospholipid bilayers either by forming a transmembrane pore or ion channel or by exhibiting surfactant activity, accompanied by the enhancement of permeability and the leakage of ions and molecules as a consequence [17,18,19]. The mode of MEL action can be influenced by the compositions of lipid membranes (electric charge or packing density) [20]. Due to its well-defined effect against the plasma membrane, MEL has consistently been used as an antimicrobial peptide (AMP). For example, Lee and Lee [21] showed that MEL has a dual antimicrobial mechanism against *Candida albicans*, disrupting target microorganism membranes and triggering apoptosis through the mitochondria/caspase-dependent pathway [21]. MEL and its derivatives and analogues also show anti-leishmanial activity in vitro. It was shown that hybrids of melittin and other AMPs cause a decrease in electrical potential and morphological changes in the membranes of *Leishmania donovani* with a fast loss of ATP (reviewed by McGwire and Kulkarni [22]). These MEL-induced morphological changes in membranes could be attributed to membrane protein aggregation, hormone secretion, and/or membrane potential alterations. Furthermore, MEL can increase the activity of several enzymes, such as G-protein, protein kinase C, adenylate cyclase, and phospholipases [23], including phospholipase A_2_ (PLA_2_). MEL also exerts potent cardioactive effects. It produces dose- and time-dependent increases in mean arterial pressure (MAP) and heart rate (HR) [24] and inhibits the activity of Na^+^K^+^-ATPase [25]. Due to the increase of the permeability of the plasma membrane to ions, especially Na^+^ and Ca^2+^, melittin causes significant morphological and functional cell changes, especially in excitable tissues, such as cardiac myocytes ([26] and references therein). Since MEL is a nonspecific cytolytic peptide that attacks all lipid membranes, leading to significant toxicity, it has been proposed that this peptide could also be used in fighting many disorders, with high therapeutic benefits [2,27]. Jeong, et al. [28] showed that melittin significantly suppresses matrix metalloproteinase-9 (MMP-9), which plays a role in atherosclerosis, and TNF-α-induced MMP-9 expression in human aortic smooth muscle cells (HASMCs). Additionally, melittin decreases the expression levels of many cytokines and growth factors in an atherosclerotic mouse model [26], affecting the processes of the immune and haematopoietic systems [29]. Melittin also inhibits transcriptional activators and simultaneously increases the expression of death receptors on the surface of cells, leading to apoptosis [30]. Due to this and many other biological actions of melittin, this polypeptide is considered a potential compound that may be useful in various medical treatments [31,32]. However, almost all studies concerning melittin bioactions have been performed on vertebrate models or cell lines. Recently, due to cheap and easy breeding and the possibility of obtaining a large number of individuals in a short time, every year, the number of examples of replacing vertebrate models in basic biomedical research by insect species has increased, especially by beetles [33]. The most important argument for this is the high level of structural and functional homology between the molecular pathways involved in the regulation of basic life processes in beetles and vertebrates [33]. For this reason, beetles may be promising model organisms and may also be of use in screening studies concerning MEL biological activities to follow the rule of the 3Rs (replacement, reduction, refinement). However, it is very important to first evaluate whether MEL acts in similar manner on beetles and vertebrate tissues.

Thus far, few attempts have been made to study the effects of bee venom and its components on invertebrates, especially insects. Galdiero et al. [34] observed the ecotoxic and genotoxic effects of MEL on *Daphnia magna*, and Söderhäll [35] found that this peptide induces rapid degranulation and the lysis of isolated granular cells and blocks the prophenoloxidase-activating system in the fresh water crayfish *Pacifastacus leniusculus*. In addition, Mitchell et al. [36] showed that MEL noncompetitively inhibits acetylcholinesterase activity in the third-instar larva of *Drosophila melanogaster*.

In this short report, we present the initial data from studies on the impact of melittin on the functioning of the immune system and the contractile activity of the heart in the *T. molitor* beetle. Because of the cheap and easy breeding, as well as the possibility of extrapolating the results of research on vertebrates [33], employing an “easier” insect model can bring benefits, such as research on a wider scale to explore many properties of melittin. For these reasons, it is valuable to examine the influence of melittin on insect physiology. We show that in the case of haemocytes, insect “blood” cells, melittin acts in a similar way as in vertebrates, and the same applies to heart activity, although the effect is marginal, which can be modulated by many different compounds [37]. Our results indicate the possibility of using this model in the early steps of pharmacological studies of new melittin-based compounds.

## 2. Results

### 2.1. Pro-Apoptotic Activity of Melittin on Haemocytes

**Figure 1:**
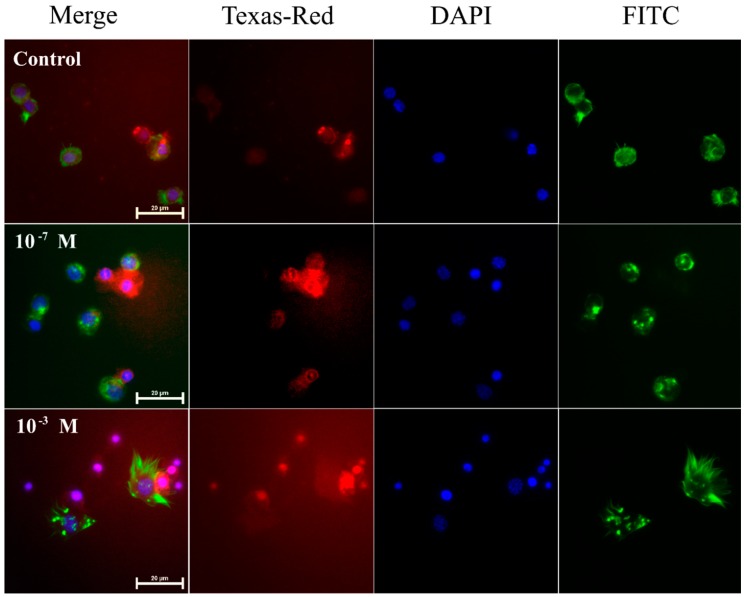
Representative fluorescence microscopic images showing induced apoptosis in haemocytes from 4-day-old male *T. molitor* beetles after an application of physiological saline (control) and melittin at concentrations of 10^−7^ M and 10^−3^ M. Merge—merged photos of the presented fluorescent channels; Texas-Red—haemocytes were stained with SR-VAD-FMK for the detection of caspase activity (red); DAPI—DNA staining (blue); FITC—haemocytes stained with Oregon Green^®^ 488 phalloidin to visualize F-actin cytoskeleton (green). The bar shows a 20 µm scale.

**Figure 2:**
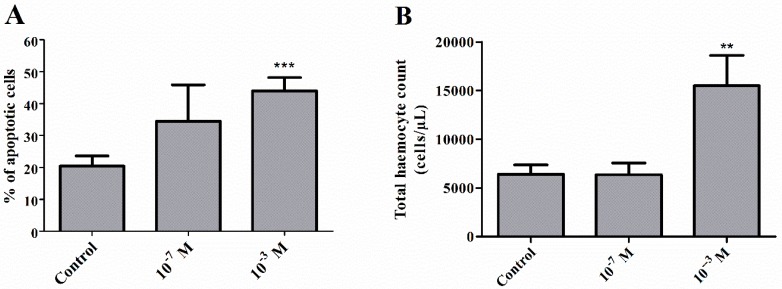
Percentage ratio of apoptotic haemocytes (**A**) and total haemocyte count (THC) in the haemolymph (**B**) of 4-day-old male *T. molitor* beetles after the application of melittin at 10^−7^ M and 10^−3^ M concentrations. Values are presented as the mean ± SEM; **, *p* ≤ 0.01, ***, *p* ≤ 0.01.

Injection of melittin into 4-day-old males of *T. molitor* beetles resulted in an increased number of apoptotic cells in the haemolymph (Figure 1 and Figure 2A). The increase in the number of apoptotic haemocytes showed a dependence on the dose, increasing by 14.03% at a concentration of 10^−7^ M (Student’s *t*-test, *t* = 1.183; *p* = 0.276) and by 24.02% at a concentration of 10^−3^ M (Student’s *t*-test, *t* = 4.545, *p* ≤ 0.001) compared to the control level of 20% of cells.

### 2.2. Total Haemocyte Count

Measurements of the total haemocyte count (THC) showed statistically significant differences between the groups in the number of cells (Kruskal‒Wallis test, *H* = 9.402, *p* ≤ 0.01). Interestingly, the changes were reported only after administration of melittin at a concentration of 10^−3^ M. Compared to the control, these differences were statistically significant (Student’s *t*-test, *t =* 3.057, *p* ≤ 0.01) (Figure 2B).

### 2.3. Phagocytic Assay

**Figure 3:**
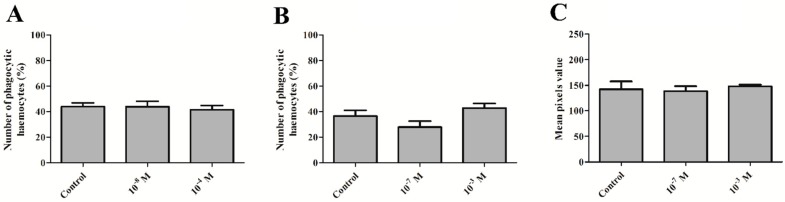
Percentage of phagocytic haemocytes after the direct application of melittin (**A**) and at 24 h after the injection of melittin (**B**), as well as the phenoloxidase (PO) activity (**C**) in the haemolymph of 4-day-old male *T. molitor* beetles at 24 h after the application of peptide. Direct application—haemocytes collected from non-injected individuals; the direct effect was examined by adding melittin to incubation solution at a concentration corresponding to the polypeptide concentration in beetle haemolymph at a 10^−7^ M and 10^−3^ M dilution (final concentration 10^−8^ M and 10^−4^ M). PO activity is based on the mean pixel value of the images. Means ± SEM are given, *n* ≥ 8.

In the case of the phagocytic bioassay, no statistically significant changes were noted. No changes in the number of haemocytes involved in phagocytosis were observed during the direct effect of melittin on insect haemocytes and 24 h after melittin injection (one-way ANOVA, *F* = 0.185, *p* = 0.833 and *F* = 2.967, *p* = 0.079) (Figure 3A,B).

### 2.4. Phenoloxidase Activity

The activity of phenoloxidase in the haemolymph of male *T. molitor* beetles did not change at 24 h after injections of melittin at concentrations of 10^−7^ and 10^−3^ M (Kruskal‒Wallis test, *H* = 5.146, *p* = 0.76) (Figure 3C).

### 2.5. Heart Assay

**Figure 4:**
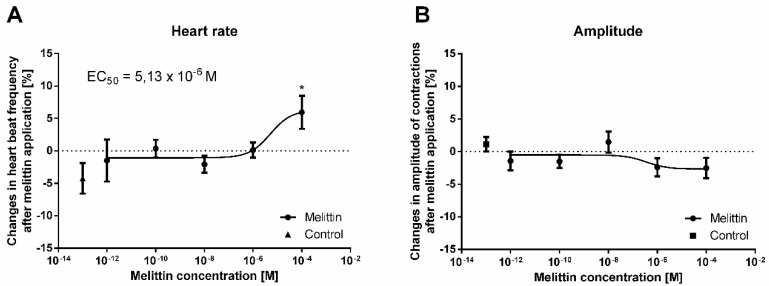
Percentage changes in the mean frequency (**A**) and amplitude (**B**) of heart contractions in *T. molitor* compared to the control after melittin application. Means ± SEM are given for *n* = 6. Significant differences from the control (saline application) are indicated by *, *p ≤* 0.05, (one-way ANOVA test).

In the bioassay, we tested the effects of melittin on the contractile activity of the myocardium of the *T. molitor* beetle at a concentration range from 10^−12^ M to 10^−4^ M. Changes in the amplitude (inotropic effect) and frequency of contractions (chronotropic effect) were measured. Under control conditions, during continuous perfusion with physiological saline (PS), the average heart rate was 86.2 beats per minute. The application of an additional 10 µL of PS did not cause any significant changes in the heart rhythm. Additionally, the application of melittin at a concentration range from 10^−12^ M to 10^−6^ M did not cause any significant difference in the contractile activity. However, when MEL was applied at 10^−4^ M, the heart rate increased (positive chronotropic effect) by approximately 9% compared to the control, leaving the amplitude of contractions at the same level (Figure 4). This effect was slight but statistically significant (one-way ANOVA, *F* = 2.594; *p* ≤ 0.001) compared to the control. The calculated EC_50_ value for melittin was 5.13 × 10^−6^ M.

## 3. Discussion

In this report, for the first time, we describe the immunotropic and cardiotropic activity of melittin in heterologous bioassays with the beetle *T. molitor*, a model insect species. The present research is a preliminary study that may indicate the direction of more complex future studies on the impact of melittin on insect physiology.

Many cytotoxic activities of melittin are associated with the pro-apoptotic properties of this polypeptide. Apoptosis, known as programmed cell death, is a key process associated with many cellular events, and melittin-induced apoptosis has been extensively studied. This peptide leads to the apoptosis of various cell types, including erythrocytes and leukocytes, as well as the full spectrum of cancer cells [27,38]. The pro-apoptotic action of melittin was also observed in arthropod cells. Söderhäll [35] showed that melittin induced the degranulation and lysis of granular cells isolated from crayfish *P. leniusculus*. Our study also revealed that melittin induces apoptosis in *Tenebrio* haemocytes. Interestingly, this effect was only observed when MEL was injected at a concentration of 10^−3^ M. Moreover, the apoptotic response was correlated with a simultaneous increase in the number of haemocytes in the haemolymph. This effect is likely related to the mobilization of haemocytes adhered to insect tissues [39]. A possible explanation may be related to compensation for the high ratio of apoptotic cells, which was observed after the application of melittin at a concentration of 10^−3^ M. Simultaneously, in a comparative experiment, we examined the cytotoxic activity of melittin at a concentration range of 10^−9^–10^−5^ M on human red blood cells, the most numerous cells in the circulatory system, to confirm the literature data [29,39] on the haemolytic activity of this compound. We observed that the threshold of the haemolytic activity of this peptide on erythrocytes began at a concentration of 10^−8^ M (results not shown). The results regarding the effect of melittin on the number of haemocytes and their apoptosis closely correspond with the phagocytic response of haemocytes. The percentage of haemocytes involved in the phagocytosis of latex beads was not changed, regardless of the time of cell contact with melittin. Similar results were obtained by Lee et al. [40], who showed that melittin administration did not affect the phagocytic activity of mouse bone marrow-derived macrophages (BMDMs). The results of the phagocytic assay support our hypothesis about the compensation of the pro-apoptotic action of melittin on insect haemocytes by mobilization of an additional pool of these cells to haemolymph. This mechanism likely ensures the activity of the cellular response at the appropriate level.

One of the most important mechanisms that connect cellular and humoral responses in arthropods is the activity of phenoloxidase (PO) [41]. This enzyme participates not only in cuticle melanisation but also in the pathogen recognition process [42]. The research conducted on the crayfish *P. leniusculus* showed that melittin may reduce the activity of this enzyme in arthropod haemolymph, but only during the co-injection with laminarin, a stimulator of arthropod immune system activity [35]. As in crayfish, a single injection of melittin in the beetle *T. molitor* did not lead to changes in PO activity. Generally, melittin influences the humoral response of vertebrates and acts as an anti-inflammatory compound by suppressing innate immune signalling pathways, including Toll-like receptors 2 and 4 (TLR2, TLR4), CD14, Nuclear factor-kappa B Essential Modulator (NEMO), and Platelet-Derived Growth Factor Receptor β (PDGFRβ) [38,43]. This immunosuppressive action of melittin leads to a reduction in the inflammatory process in various tissues and organs, such as joints, skin, or neuronal tissue [38]. Moreover, melittin can inhibit the innate humoral response during infection. Research by Park et al. [44] showed that melittin suppresses the release of prostaglandin E2 (PGE2) and nitric oxide (NO) from lipopolysaccharide (LPS)-treated RAW264.7 cells. On the other hand, much evidence indicates the pro-inflammatory action of this polypeptide [45]. For example, the expression of genes encoding TNF-α, IL-1, and IL-6 cytokines in PMA-differentiated U937 cells was increased after the co-administration of melittin and LPS compared to LPS stimulation alone [45,46]. For these reasons, the next step will be to evaluate the role of melittin in the modulation of the insect immune system during pathogen infection, including the specificity of the effect of MEL on the molecular pathways associated with the insect immune response. Due to the high level of homology at the molecular level between insect and vertebrate innate immune responses, confirmation of a similar mode of action of melittin on immune-related pathways, such as Toll, IMD, or JAK/STAT, may be useful for searching an alternative model organism in screening research concerning MEL biomedical applications [33].

No less important are the results of melittin influence on insect heart contractile activity. As in the previous section regarding the immune system, our knowledge about the action of melittin on the heart is based only on research conducted on the vertebrate model. Because melittin directly influences Ca^2+^ influx and Na^+^ channels in excitable tissues, this polypeptide affects the action of the vertebrate heart [47]. For example, Brovkovich and Moibenko [48] showed that immediately after application, melittin causes an increase in the contractility of the isolated papillary muscle of the rat heart. After this phase, a dose-dependent decrease in the strength of heart contractions was noted [48]. The bimodal effect of melittin application was also reported in research performed on the isolated guinea pig atria. In this study, at a concentration of 0.1–0.8 μmol/dL, melittin enhanced the contraction of the left atria. However, the use of melittin at higher concentrations (1.6–12.8 μmol/dL) led to the suppression of atrial contractions. Additionally, at a concentration of 0.1–30 μmol/dL, melittin increased the frequency of contractions of isolated right atria [49]. However, there is also evidence of the irreversible paralysis of isolated rat hearts after using 20–40 μg of melittin [50].

In our study, the application of melittin at a concentration of 10^−4^ M to the semi-isolated heart of the *Tenebrio* beetle caused a slight (approximately 9% compared to the control) but significant positive chronotropic effect without simultaneous inotropic changes in the contractile activity of the myocardium. Due to the already known action of MEL on ion channels in vertebrates, a weak *Tenebrio* heart response after the application of this polypeptide may indicate some species-specific activity. Interestingly, whole bee venom caused the strong stimulation of *Tenebrio* heart contractility, which also led to cardiac arrest during the systolic phase (unpublished data). This result suggested that other compounds identified in bee venom may be crucial for exerting a positive chronotropic effect. This effect may be related to the action of tertiapin, which selectively blocks G-protein–gated inwardly rectifying K^+^ channel (IK_Ach_) [51]. However, we did not exclude the synergistic effect of various components of bee venom. For this reason, in our future research, we will evaluate the cardiotropic properties and potential synergistic actions of other main components of bee venom.

In summary, the presented results clearly showed that melittin affects basic insect immune mechanisms and elicits effects resembling the reactions observed in vertebrate models. These results may be useful for identifying new models for screening biomedical research regarding the influence of melittin and its derivatives on innate immune mechanisms. For this reason, the continuation of the presented work and the determination of how melittin acts on, homological to vertebrates, insect immune-related molecular pathways, such as Toll, IMD, or JAK/STAT, are crucial. Further research on the effect of melittin activity on insect physiology is also necessary due to the different responses of insect hearts compared to vertebrate hearts to melittin application. An explanation of the physiological and biochemical basis of this phenomenon and further research concerning other components of bee venom may be important for identifying new anaphylactic substances.

## 4. Materials and Methods

### 4.1. Insects

In all bioassays, adult males of the mealworm beetle *Tenebrio molitor* were used. Individuals were obtained from the culture maintained at the Department of Animal Physiology and Development at Adam Mickiewicz University in Poznań (AMU) according to a method described by Rosinski [52]. To avoid immune-senescence and sex-specific differences in immune system activity, all bioassays concerning this part of the present study were conducted on 4-day-old males. In the heart assay, 30-day-old individuals were used. Although the EU legislation on care and use of experimental animals does not require any permits from the ethics committee, all insects were handled carefully, and the number of animals used was the minimum necessary to achieve the aims of this study.

### 4.2. Melittin Injection and Haemolymph Collection

Before the injection of melittin (Sigma-Aldrich, St. Louis, MO, USA) and haemolymph collection, individuals were anaesthetized with endogenous CO_2_. Then, the beetles were disinfected with 70% ethanol (Avantor Performance Materials Poland S.A., Gliwice, Poland) and washed with distilled water.

The beetles were injected with 2 µL of melittin (Sigma-Aldrich, St. Louis, MO, USA) at concentrations of 10^−7^ and 10^−3^ M or 2 µL of physiological saline (PS; 274 mM NaCl, 19 mM KCl, 9 mM CaCl_2_) as a control. Injections were made with a Hamilton syringe (Hamilton Co., Reno, Nevada, USA) on the ventral side of the body of the beetles under the third pair of legs, inserting the needle towards the head. The melittin concentration used was based on literature data on the concentration of this polypeptide in a single dose of venom applied by bees (10^−4^ M) [53] and the threshold value for melittin-induced haemolysis (10^−8^ M) [30,54]. Due to the average volume of 20 µL of haemolymph in adult *Tenebrio molitor* beetles and the injection of 2 µL of melittin solution, the concentration of this polypeptide in the haemocoel was ten-fold lower [55].

The haemolymph was collected by cutting the tibia of the first pair of legs.

### 4.3. Apoptosis

To detect active caspases in haemocytes, the sulforhodamine derivative of valyl alanyl aspartic acid fluoromethyl ketone, a potent inhibitor of caspase activity (SR-VAD-FMK, Enzo Life Sciences, Inc., New York, NY, USA) was used according to the manufacturer’s instructions. Haemocytes were allowed to adhere for 30 min and then washed 3 times with PS, incubated in reaction medium (1/3 × SR-VAD-FMK) for 30 min at room temperature in the dark, washed again 3 times with wash buffer for 5 min at room temperature, and fixed in 4% paraformaldehyde for 10 min. Next, the haemocytes were permeabilized in PS containing 0.1% Triton X-100 (Sigma-Aldrich, St. Louis, MO, USA) for 10 min at room temperature and then washed again with PS. To visualize the F-actin cytoskeleton, the haemocytes were stained with Oregon Green^®^ 488 phalloidin (Invitrogen Carlsbad, CA, USA) for 20 min at room temperature in the dark. After washing with physiological saline, the haemocytes were stained with DAPI solution (Sigma-Aldrich) for 5 min at room temperature in the dark to visualize the nuclei. Finally, the preparations were washed with distilled water and mounted using mounting medium (95% glycerol with 2.5% 1,4-diazabicyclo[2.2.2]octane (DABCO) in PBS; Sigma-Aldrich, St. Louis, MO, USA). The haemocytes prepared in this way were examined using a Nikon Eclipse TE 2000-U fluorescence microscope (Nikon, Tokyo, Japan).

### 4.4. Total Haemocyte Count

The total haemocyte count was determined based on a method previously described by Urbanski et al. [56]. The samples of *Tenebrio* haemolymph (2 µL) were diluted in physiological saline containing anticoagulation buffer (4.5 mM citric acid and 9 mM sodium citrate, Sigma-Aldrich, St. Louis, MO, USA) (5:1 *v*/*v*). Then, the prepared suspension was examined with a Bürker chamber (Waldemar Knittel Glasbearbeitungs- GmbH, Braunschweig, Germany) and a Nikon PrimoStar light microscope (Nikon, Tokyo, Japan).

### 4.5. Phagocytic Assay

A phagocytic assay was performed according to the method described by Urbanski et al. [57]. Phagocytosis was conducted in vitro using fluorescently labelled latex beads (Sigma-Aldrich, St. Louis, MO, USA). The samples of haemolymph (2 µL) were incubated for 30 min with suspension of PS containing anticoagulation buffer and latex beads. The specimens were analysed using Nikon Eclipse fluorescence microscope (Nikon, Tokyo, Japan). The results are expressed as a percentage ratio of haemocytes with fully phagocytosed latex beads to the overall number of haemocytes visible on a single photo. In each biological repetition, the number of haemocytes participating in phagocytosis was estimated based on 5 random photos.

In the case of the phagocytic assay, despite melittin administration by injection, the direct effect of this compound was also tested. For this purpose, melittin was added to the suspension of latex beads with anticoagulant buffer. The melittin concentrations in this suspension corresponded to the final concentration of MEL in the insect after haemolymph injection (10^−8^ and 10^−4^ M, respectively). The haemocytes were incubated in a suspension of latex beads with the tested polypeptide for 30 min.

### 4.6. Phenoloxidase Activity

Phenoloxidase activity was analysed based on the colorimetric method described by Sorrentino et al. [58]. Haemolymph samples (1 µL) were placed on Whatman No. 52 filter paper soaked with phosphate buffer (10 mM; Sigma-Aldrich, St. Louis, MO, USA) and 3,4-Dihydroxy-DL-phenylalanine (DL-DOPA; 2 mg/ mL, Sigma-Aldrich, St. Louis, MO, USA). After 30 min of incubation, the samples were air dried and scanned using Sharp AR 153EN (600 dpi, 8 bits, grey scale; Sharp Corporation, Osaka, Japan). The PO activity was determined by measuring the intensity of colour in the central part of the obtained spots. The results were expressed as the mean pixel value.

### 4.7. Heart Bioassay

To measure the activity of melittin on semi-isolated heart preparations, the microdensitometric method, coupled with a computer-based data analysis, was used as previously described [59,60,61]. After anaesthesia, insects were decapitated, and the abdomen was removed. A cut was then made on the ventral side of the abdomen, and viscera were removed leaving only the dorsal vessel and alary muscles—semi-isolated heart preparation. Next, the preparation in incubation chamber was installed on the microdensitometer MD-100 (Carl Zeiss, Oberkochen, Germany) connected to a computer. The heart was subjected to constant perfusion with fresh PS at a rate of 140 μL/min. Melittin was applied at the injection port using a Hamilton syringe. After 15–20 min of acclimatization, the activity of the isolated heart was recorded for 120 s. The activity of the beetles’ myocardium under perfusion with PS was recorded for 30 s. Next, the tested peptide was applied, and the activity of the heart was recorded for an additional 90 s. This procedure was repeated at 5-min intervals for each concentration tested. To test whether the semi-isolated heart preparations functioned properly, the cardiostimulatory peptide proctolin was used as an internal standard. The activity of melittin was presented as a percentage change in the control frequency of heart contractions.

### 4.8. Statistical Analysis

For statistical analysis of the obtained data, we used GraphPad software ver. 6 (GraphPad Software, San Diego, CA, USA) (Department of Animal Physiology and Development AMU license). Before statistical analysis, the normality of distribution (the Shapiro‒Wilk test) and the homogeneity of variance (the Brown‒Forsythe test and the Levene test) were checked. For the analysis of groups with normal distribution, one-way ANOVA with Tukey’s post hoc or Student’s *t*-test were used. The data without normal distribution were analysed with the Kruskal‒Wallis test. Values of *p* ≤ 0.05 (*), *p* ≤ 0.01 (**) or *p* ≤ 0.001 (***) were considered statistically significant.
